# PReS-FINAL-2123: Feto-maternal outcome in patients with systemic sclerosis

**DOI:** 10.1186/1546-0096-11-S2-P135

**Published:** 2013-12-05

**Authors:** SE Maher, FM Ismail

**Affiliations:** 1 Pediatric, El Minia University, Minia, Egypt; 2 Rheumatology, El Minia University, Minia, Egypt

## Introduction

Progressive systemic sclerosis is a life threating disease typically involve the heart, lungs, and other organs. pregnancy is a stressful condition that can affect the course of the disease.

## Objectives

To study the maternal and fetal outcomes in pregnant women with Systemic Sclerosis (ssc) and to analyze the possible associated risk factors.

## Methods

Twenty pregnant women with ssc and twenty age-matched low risk pregnant women were recruited in this study. Patients were evaluated clinically and laboratory at the entry of the study and at monthly intervals. Different pregnancy outcome measures were studied. Impacts of pregnancy on scleroderma patients were determined during and after pregnancy. The possible associated risk factors were analyzed.

## Results

Twenty ssc pregnant women were recruited in this study with a mean age 29.6 ± 3. Eight (40%) of them had limited ssc, and twelve (60%) had diffuse type. Pregnancies were complicated by maternal flare of underlying disease in six (30%) pregnant patients. Six patients (30%) had preterm labor. Four patients (20%) had small for gestational age (SGA) infants, two of them (10%) had intra uterine growth retardation (IUGR). Two patients (10%), with diffuse type, fulfilled criteria of antiphospholipid syndrome (APS) but unfortunately the pregnancy ended in miscarriage. Eight (40%) full-term infants were born two of them were SGA, 2 cases with miscarriage due to renal crisis and pulmonary hypertension and another two cases with intra uterine fetal death (IUFD). The live birth rate was 14/20 (70%) in ssc group.

## Conclusion

Women with ssc can safely have healthy pregnancies if pregnancy is planned when the disease is stable and managed by a multidisciplinary team during pregnancy.

## Disclosure of interest

None declared.

